# Proteomics and transcriptomics combined reveal specific immunological markers in autoimmune thyroid disease

**DOI:** 10.3389/fimmu.2024.1531402

**Published:** 2025-01-13

**Authors:** Xia Chen, Hui Chen

**Affiliations:** ^1^ The Second Hospital & Clinical Medical School, Lanzhou University, Lanzhou, China; ^2^ Department of Endocrinology and Metabolism, The Second Hospital & Clinical Medical School, Lanzhou University, Lanzhou, China

**Keywords:** hyperthyroidism, hypothyroidism, ISG15, ZNF683, IGHG3

## Abstract

**Objective:**

The pathogenesis of AITD remains unclear to date. This study employs a combination of proteomics and transcriptomics analysis to identify and validate specific immune response markers in patients with hyperthyroidism and hypothyroidism, thereby providing a scientific basis for the clinical diagnosis and treatment of AITD.

**Methods:**

By collecting serum and whole blood tissue samples from patients with hyperthyroidism, hypothyroidism, and healthy controls, this study utilizes a combination of transcriptomics and proteomics to analyze changes in immune-related signaling molecules in patients. Specific biomarkers were identified, and the ELISA method was employed to determine the expression levels of these clinical markers and their correlation with clinical features of the patients, ultimately establishing a predictive model.

**Results:**

Transcriptomic and proteomic analyses were conducted to identify differentially expressed genes and proteins in patients with hyperthyroidism and hypothyroidism compared to healthy controls. Enrichment analysis revealed that these differentially expressed genes and proteins are primarily associated with immune function, antigen-antibody binding, and alterations in immune cells. Through the combined analysis of transcriptomics and proteomics, key genes IGHG3, ISG15, and ZNF683 were identified. ELISA results from clinical patient serum samples indicated that the levels of IGHG3 were significantly higher in both the hyperthyroid and hypothyroid groups compared to the control group (P<0.05). Additionally, the serum levels of ISG15 in the hyperthyroid group were greater than those in both the control and hypothyroid groups (P<0.05), while the serum levels of ZNF683 in the hypothyroid group exceeded those in the control and hyperthyroid groups (P<0.05). Furthermore, all three biomarkers correlated with the thyroid function of the patients. Prediction models for hyperthyroid and hypothyroid patients were constructed using IGHG3, ISG15, and ZNF683, demonstrating good performance metrics and decision effect.

**Conclusion:**

In patients with hyperthyroidism and hypothyroidism, significant changes primarily occur in immune function and immune cells when compared to healthy individuals. Key signaling molecules were identified: ISG15 for hyperthyroidism, ZNF683 for hypothyroidism, and IGHG3 common to both conditions. These findings provide new biomarkers for the diagnosis and monitoring of clinical patients, thereby offering a scientific basis for research on AITD and personalized treatment approaches.

## 1 Introduction

Autoimmune thyroid diseases (AITD) account for the majority of thyroid diseases. Its pathogenesis is the result of interaction of many factors, including genetic factors, environmental factors and so on. It is characterized by the loss of self-tolerance to thyroid antigen. AITD primarily include Graves’ disease (GD) and Hashimoto’s thyroiditis (HT). There are two types of disease exhibit two extreme clinical manifestations: hyperthyroidism and hypothyroidism (1). In Graves’ disease, the loss of immune tolerance leads to immune-mediated infiltration of T lymphocytes and activation of TSH receptor (TSHR)-reactive B cells. This process eventually produces autoantibodies to TSHR, which stimulate thyroid cell growth and secretion, causing hyperthyroidism, goiter, as well as associated conditions such as ophthalmopathy and dermopathy (2). Hashimoto’s thyroiditis shares similar humoral mechanisms with Graves’ disease. In HT, the patient’s body automatically reacts to produce antibodies to thyroid peroxidase (TPO) and thyroglobulin (Tg) (3), along with predominantly blocking TSHR antibodies. This combination triggers an autoimmune response characterized by a high inflammatory burden, leading to apoptosis and necrosis of thyroid cells, ultimately resulting in hypothyroidism. Currently, it is believed that the pathogenesis of AITD is associated with factors such as immune regulation, genetics, environmental variables, gender, and epigenetics. However, the molecular mechanisms through which alterations in immune function led to changes in thyroid tissue remain largely unexplained. The states of hyperthyroidism and hypothyroidism in AITD patients can cyclically convert or appear interchangeably (4, 5). Some individuals with typical Graves’ disease, particularly those with autoimmune ophthalmopathy, may experience episodes of hypothyroidism that necessitate levothyroxine replacement therapy. Similarly, Hashimoto’s thyroiditis patients may persistently exhibit hyperthyroidism along with associated conditions like ophthalmopathy and dermopathy (6). This implies that different types of TSHR antibodies in patients with AITD may undergo transformation, akin to the two sides of a coin, where hyperthyroidism can convert to hypothyroidism and vice versa. Consequently, the timely differentiation between hyperthyroid and hypothyroid patients presents challenges in clinical practice. Currently, the potential molecular mechanisms underlying the heterogeneous clinical manifestations in these patients remain unclear.

In clinical practice, monitoring changes in thyroid function mainly relies on serological tests, through testing thyroid function index and antibodies in the diagnosis of disease, In particular, the changes of the disease are judged by the detection of TSH receptor (TSHR) autoantibodies (TRAb). At present, the detection methods of TRAb mainly include binding assay and bioassay, and binding assay can only be used to detect total TRAb. The IMMULITE^®^ 2000/2000XPi TSI method can diagnose and monitor the progression of GD through the detection of thyroid-stimulating immunoglobulins (TSI). However, it is still unable to differentiate between the various types of TRAb. Bioassays, which can determine the different types of TRAb, have not been widely used in clinical practice due to their long and cumbersome operation time. This limitation poses challenges for timely prediction and differential diagnosis of disease progression. Currently, antithyroid medications remain the first-line treatment, while traditional therapeutic approaches such as surgery and radioactive iodine therapy also come with certain side effects, complicating the clinical management of AITD (7). Accurate diagnosis and individualized treatment of patients with hyperthyroidism and hypothyroidism present significant challenges. Therefore, elucidating the molecular mechanisms underlying AITD and identifying key factors related to its onset, progression, and changes in clinical status is critically important. This study aims to identify specific immune response biomarkers that differentiate between healthy person and hyperthyroidism or hypothyroidism through whole blood transcriptomic analysis combined with serum Data-independent acquisition (DIA) proteomic analysis. We will utilize HPLC-QTOF/MS technology to characterize the original components of the blood. Additionally, we will employ serum tandem mass tag (TMT) quantitative proteomic techniques to obtain differentially expressed proteins (DEPs). Using the ELISA method, we will identify the specific expression of immune response-related biomarkers in clinical patients, analyze the correlation between basic characteristics of different clinical patients and immune markers, and explore potential molecular mechanisms behind the onset and progression of AITD. This research in order to provide a scientific basis for the diagnosis and individualized treatment of AITD.

## 2 Materials and methods

### 2.1 Sample collection

Serum and whole blood tissue samples were collected from patients diagnosed with AITD-associated hyperthyroidism and hypothyroidism (new diagnosis and untreated), non-AITD hyperthyroidism and hypothyroidism patients (new diagnosis and untreated), as well as healthy controls, between January 2023 and January 2024 at the Second Hospital of Lanzhou University. Diagnosis was made according to the guidelines (8), and individuals with comorbidities, extra-thyroidal manifestations, or those receiving treatment with medications such as steroids that may affect immune function or thyroid function were excluded from the study. All participants were required to fast for 8 hours before blood collection. The blood samples used for sequencing were placed in liquid nitrogen for 24 hours and subsequently stored at -80°C to ensure sample stability. In addition to the biological samples, relevant clinical data from the patients were gathered, including gender, age and body mass index (BMI), and laboratory test results for total triiodothyronine (TT3), total thyroxine (TT4), free triiodothyronine (fT3), free thyroxine (fT4), thyroid-stimulating hormone (TSH), thyroglobulin (Tg), anti-thyroglobulin antibody (TgAb), thyroid peroxidase antibody (TPOAb), TRAb. The serum levels of TT3, TT4, fT3, fT4, TSH, TRAb, Tg, TgAb and TPOAb were measured using reagent kits from Siemens USA, employing a chemiluminescent analysis method. The reference ranges for these parameters are as follows: TT3 (1.01-2.95 nmol/L), TT4 (55.4-161.25 nmol/L), fT3 (2.77-6.31 pmol/L), fT4 (10.44-24.38 pmol/L), TSH (0.38-4.34 µIU/ml), TG (negative value reference range: 0.83-68 ng/ml), TgAb (negative value reference range: 0-4.5 IU/ml), and TPOAb (negative value reference range: 0-60 U/ml). Additionally, TRAb was measured using a reagent kit from Xinchanye Biotechnology Co., Ltd., utilizing the same chemiluminescent technique, with a negative value reference range of 0-1.5 IU/L. The study was conducted in accordance with the ethical principles of the Declaration of Helsinki. The study was approved by the hospital Medical Ethics Committee (approval number:2023A-802). The basic information of the participants is provided in the **Table 1**.

**Table 1:** The basic information of the participants.

	Healthy Control (n=60)	AITD hyperthyroidism (n=127)	AITD hypothyroidism (n=66)	non-AITD hyperthyroidism (n=31)	non-AITD hypothyroidism (n=26)
Gender					
female	32	69	35	18	15
male	28	58	31	13	11
Age (year)					
	35.83 ± 9.56	34.40 ± 10.66	37.34 ± 11.78	36.75 ± 12.91	35.11 ± 12.76
BMI (kg/m²)					
	22.15 ± 3.24	21.52 ± 4.05	22.88 ± 3.95	21.93 ± 3.03	22.61 ± 2.63

### 2.2 Transcriptome sequencing

RNA was extracted from whole blood using the TRIzol method, and the purity, concentration, absorbance peaks, and integrity of the extracted RNA were evaluated. Quality control of the raw data was performed using fastp (v0.20.0). Once the samples passed quality control, library construction was initiated. The StringTie (stringtie-2.1.3b.Linux_x86_64) software was used to merge the reconstruction results from all samples, yielding an optimized transcript structure annotation file. Subsequently, the library was validated. After successful validation, sequencing was performed using the Illumina NovaSeq 6000. The results were processed using Ballgown (R script) to obtain the gene expression readcount matrix. Differential analysis was conducted using the readcount matrix to identify differentially expressed genes between sample groups. Differential genes were annotated using BLASTALL (v2.2.26, with an e-value set to 1e-5) for functional analysis, including GO and KEGG pathway annotations.

### 2.3 Serum proteomics analysis

Proteins were extracted from biological samples for analysis. Data-independent acquisition (DIA) mass spectrometry was employed for data collection, obtaining full scan information for all precursor ions and their corresponding fragment ions. The output files generated by the Thermo Scientific Q Exactive HF mass spectrometer were then used to identify differentially expressed proteins. The raw data files obtained from the Zeno-TOF 7600 (SCIEX) were imported into DIA-NN software for database search. The proteins were annotated using BLASTALL (v2.2.26, with an e-value set to 1e-5) for functional analysis, including Gene Ontology (GO) and Kyoto Encyclopedia of Genes and Genomes (KEGG) pathway annotations. Enrichment analysis of the identified differentially expressed proteins was performed, and interaction networks were constructed to elucidate their biological significance and potential roles under the experimental conditions. The clustering heatmap of all proteins was generated using the pheatmap package in R (V3.6.2). Principal component analysis (PCA) was performed using the prin_comp_data function in R (V3.6.2), and a PCA plot was created. Additionally, subcellular localization of the differentially expressed proteins was determined using the web tool available at http://cello.life.nctu.edu.tw/.

### 2.4 Joint analysis of transcriptome and proteome

A joint analysis was conducted based on the results from both transcriptomic and proteomic studies. Based on the results of transcriptomic and proteomic differential analysis, the top 50 differential genes (the 50 genes with the smallest FDR values) and the top 50 differential proteins (the 50 proteins with the largest FC values) were selected. Using the selected differential genes and proteins, the Spearman correlation between the two was calculated using the psych package in R (v3.6.2). The correlation results were further filtered with a correlation coefficient greater than 0.9 and a p-value smaller than 0.001. Based on the filtered correlation results, a differential gene-differential protein network regulatory map was constructed using Cytoscape software (v3.7.2).

### 2.5 Enzyme-linked immunosorbent assay

ISG15, ZNF683 and IGHG3 ELISA kit (Shfksc, China) were used to detect ISG15, ZNF683 and IGHG3 in human serum, respectively. According to the kit instructions, first of all, standard samples and test samples were incubated to allow binding with antibodies coated on an enzyme-linked plate. After this initial incubation, the plate was washed, and a primary antibody was added to enable specific binding with the standards and samples captured by the coated antibodies. Following another wash step, horseradish peroxidase (HRP)-labeled streptavidin was introduced to bind with biotin. Afterward, using TMB to promote colorimetric reaction. Finally, a stopping solution is added to end the reaction. The OD450 values of each reaction hole were determined. By plotting the standard sample data on a standard curve, the concentration of the target analyte in the test samples could be accurately calculated.

### 2.6 Statistical analysis

The statistical software was GraphPad Prism 8.0 and SPSS 22.0 for normally distributed continuous data, results were expressed as Mean ± SD, and comparisons between groups were performed using t-tests. Categorical data were represented by absolute numbers or percentages, with group comparisons assessed using χ² test. Additionally, logistic regression was employed to construct clinical prediction models, and ROC curves were generated to evaluate the performance of these models. *P*<0.05 was considered as significant difference. This approach ensured a robust assessment of the data collected during the study, facilitating meaningful conclusions based on statistical evidence.

## 3 Results

### 3.1 Differential gene screening

**Figure 1:**
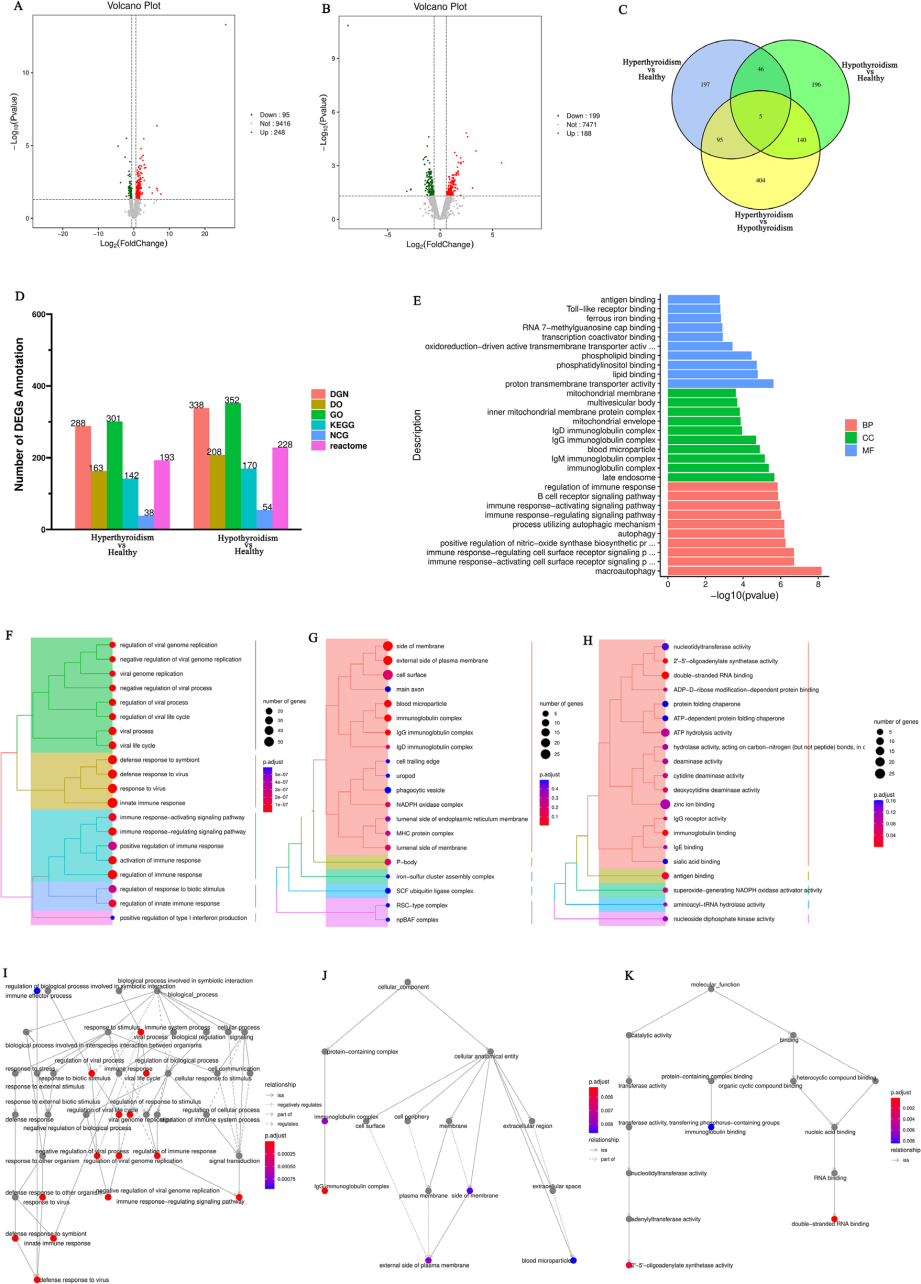
**(A)** Volcano plot of differentially expressed genes comparing the hyperthyroid group to the normal group; **(B)** Volcano plot of differentially expressed genes comparing the hypothyroid group to the normal group. Each point in the volcano plot represents a gene, with the x-axis indicating the logarithmic value of the fold change in expression between the two samples, and the y-axis representing the negative logarithmic value of the p-value; **(C)** Venn diagram of differentially expressed genes, where each circle represents a set of differential analysis combinations, and the numbers within the circles denote the count of differentially expressed genes for each combination; **(D)** Bar chart of annotation statistics for differentially expressed genes comparing the hyperthyroid group to the normal group. The x-axis represents the grouping information, while the y-axis indicates the count. Different colors denote the names of the annotation databases; **(E)** GO annotation classification statistics chart for differentially expressed genes comparing the hyperthyroid group to the normal group. The x-axis depicts the -log10(p-value), and the y-axis represents the GO classifications, with the same color indicating the same category; **(F)** GO annotation BP hierarchical diagram for differentially expressed genes comparing the hyperthyroid group to the normal group; **(G)** GO annotation CC hierarchical diagram for differentially expressed genes comparing the hyperthyroid group to the normal group; **(H)** GO annotation MF hierarchical diagram for differentially expressed genes comparing the hyperthyroid group to the normal group. In the GO annotation hierarchical diagrams, the branches of the tree represent specific terms, with branches of the same color clustered together into the same category. the color of the nodes indicates p.adjust values, representing the significance level of enrichment. and the size of the nodes reflects the count of differentially expressed genes within that term. The top 20 categories are displayed based on the minimum p.adjust values; **(I)** Interaction diagram of GO annotation BP for differentially expressed genes comparing the hyperthyroid group to the normal group. **(J)** Interaction diagram of GO annotation CC for differentially expressed genes comparing the hyperthyroid group to the normal group. **(K)** Interaction diagram of GO annotation MF for differentially expressed genes comparing the hyperthyroid group to the normal group. In the interaction diagrams of GO annotations, the colors represent p.adjust values, with points denoting different terms. The term “is a” indicates subclasses of the specified term. “negatively regulates” denotes that the term before the arrow inhibits the function of the term after the arrow. “part of” signifies that Term A before the arrow is a part of the term following the arrow, indicating a containment relationship. and “regulates” implies that the current term modulates the function of another term, which may either be promoting or inhibiting. The default parameters are set such that p-value is less than 0.01 and p.adjust is less than 0.05.

Differentially expressed genes (DEGs) selection was execution using the DESeq2 software with the following criteria: an absolute Log_2_<0.58 and *P*<0.05. This analysis identified differentially expressed genes (DEGs) between patients hyperthyroid and healthy person, revealing the sum of all 248 upregulated genes and 95 downregulated genes (**Figure 1A**). In addition, differential expression analysis was conducted between hypothyroid patients and healthy controls, resulting in the identification of 188 upregulated genes, 199 downregulated genes (**Figure 1B**). To further illustrate the relationships among these DEGs, Venn diagrams were created for each comparison group, showcasing the unique and shared DEGs among the groups (**Figure 1C**).

### 3.2 Differential gene analysis

Following the identification of differential genes, functional annotation was performed on these genes. **Figure 1D** presents a statistical overview of the number of DEGs across three groups: hyperthyroidism group, hypothyroidism group and healthy controls.

**Figure 2:**
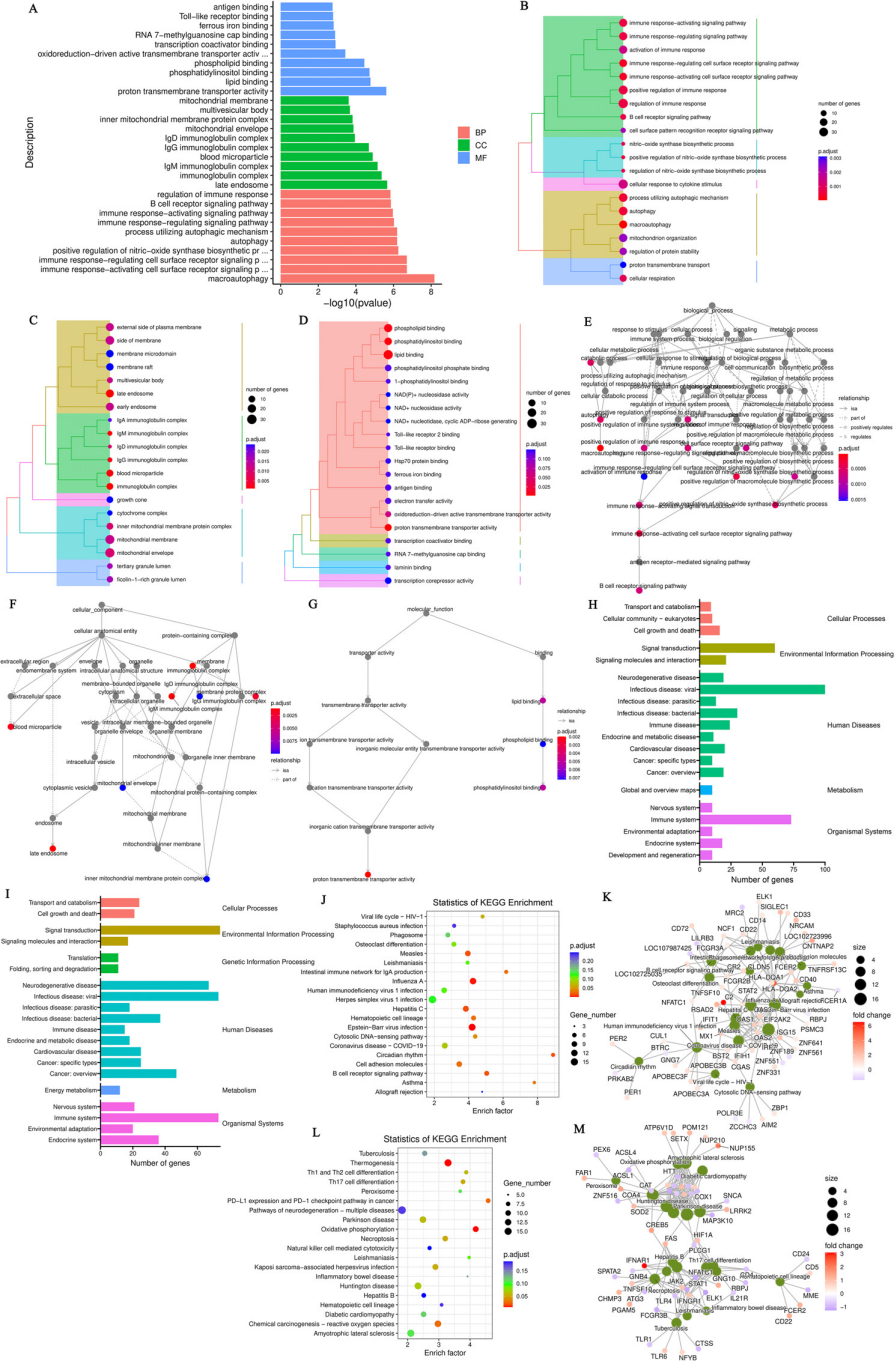
**(A)** GO annotation classification statistics chart for differentially expressed genes comparing the hypothyroid group to the normal group. The x-axis represents the -log10(p-value), while the y-axis indicates the GO classifications, with identical colors representing the same category; **(B)** GO annotation BP hierarchical diagram for differentially expressed genes comparing the hypothyroid group to the normal group; **(C)** GO annotation CC hierarchical diagram for differentially expressed genes comparing the hypothyroid group to the normal group; **(D)** GO annotation MF hierarchical diagram for differentially expressed genes comparing the hypothyroid group to the normal group. In the hierarchical diagrams of GO annotations, the branches of the tree represent specific terms, with branches of the same color clustered into the same category. The color of the nodes indicates p.adjust values, which reflect the significance level of enrichment, while the size of the nodes reflects the number of differentially expressed genes within that term. Each classification displays the top 20 categories based on the minimum p.adjust values; **(E)** Interaction diagram of GO annotation BP for differentially expressed genes comparing the hypothyroid group to the normal group; **(F)** Interaction diagram of GO annotation CC for differentially expressed genes comparing the hypothyroid group to the normal group; **(G)** Interaction diagram of GO annotation MF for differentially expressed genes comparing the hypothyroid group to the normal group. In these interaction diagrams of GO annotations, the colors represent p.adjust values, while points denote different terms. The term “is a” indicates subclasses of the specified term. “negatively regulates” denotes that the term before the arrow inhibits the function of the term after the arrow. “part of” signifies that Term A before the arrow is part of the term following the arrow, indicating a containment relationship. and “regulates” implies that the current term modulates the function of another term, which may either be promoting or inhibiting. The default parameters are set such that p-value is less than 0.01 and p.adjust is less than 0.05; **(H)** KEGG classification chart for differentially expressed genes comparing the hyperthyroid group to the normal group; **(I)** KEGG classification chart for differentially expressed genes comparing the hypothyroid group to the normal group. The left vertical axis lists the names of the KEGG secondary metabolic pathways, while the right vertical axis lists the names of the KEGG primary metabolic pathways. The horizontal axis indicates the number of genes annotated to each pathway; **(J)** KEGG pathway enrichment scatter plot for differentially expressed genes comparing the hyperthyroid group to the normal group; **(L)** KEGG pathway enrichment scatter plot for differentially expressed genes comparing the hypothyroid group to the normal group. In the scatter plots, each point represents a specific KEGG pathway, with the x-axis indicating the number of differentially expressed genes associated with that pathway and the y-axis representing the significance of enrichment, which may be indicated by p-values or other relevant metrics. Each row in the KEGG pathway enrichment scatter plots represent a specific KEGG pathway. The x-axis indicates the enrichment factor, which is the ratio of the proportion of differentially expressed genes annotated to that pathway to the proportion of all genes annotated to the same pathway. A larger enrichment factor indicates a more significant level of enrichment of differentially expressed genes within that pathway. In the scatter plots, the color of each point represents the p.adjust value, reflecting the significance level of enrichment, while the size of the points corresponds to the number of differentially expressed genes annotated to that specific pathway; **(K)** Interaction diagram of KEGG annotated pathways for differentially expressed genes comparing the hyperthyroid group to the normal group; **(M)** Interaction diagram of KEGG annotated pathways for differentially expressed genes comparing the hypothyroid group to the normal group. In these diagrams, pathways are represented as green nodes, with the size of the pathway nodes corresponding to the number of genes annotated within that pathway. Genes are represented as nodes colored from blue to red, with the color indicating the log2 fold change values of the genes. The diagrams display the top 20 pathways with the lowest p.adjust values, highlighting the most significantly enriched pathways related to differential gene expression in each condition.

The DEGs between hyperthyroidism group and healthy control group was analyzed by GO. The results of enrichment analysis show that the distribution of DEGs across three primary branches of the GO annotation system: Biological Process (BP), Cellular Component (CC), and Molecular Function (MF). The outcome revealed that the DEGs were primarily enriched in BP, including innate immune response, adjustment of immune response, response to virus, 2’-5’ oligoadenylate synthetase activity and immune response-activating signaling pathway. In terms of CC, the DEGs showed significant enrichment in cell surface, immunoglobulin complex, IgG immunoglobulin complex, NADPH oxidase complex and external side of plasma membrane. For MF, the key enrichments included antigen binding, immunoglobulin binding, IgG receptor activity (**Figures 1E–H**). In addition to the initial analysis, further interaction analyses were conducted on the DEGs within the categories of BP, CC, and MF. The results of these interaction analyses are presented in **Figures 1I–K**. For the comparison between hypothyroidism group and healthy controls, GO annotation clustering analysis was performed. The enrichment analysis indicates that the hypothyroid group DEGs were mainly enriched in several BP, including adjustment of immune response, B cell receptor signaling pathway, process utilizing autophagic mechanism and immune response-activating signaling pathway. In terms of CC, significant enrichments were observed in immunoglobulin complex and IgG immunoglobulin complex. For MF, the DEGs showed notable enrichment in proton transmembrane transporter activity and lipid binding in **Figures 2A–D**. The interaction analysis of the DEGs from the hypothyroidism group compared to healthy controls is depicted in **Figures 2E–G**.

To further elucidate the functions of the DEGs, KEGG pathway annotation was performed. The DEGs were categorized according to the types of pathways in the KEGG database. The enrichment analysis for the KEGG pathways comparing the hyperthyroidism group with healthy controls it was found that DEGs was mainly concentrated in several key points, including signal transduction, signaling molecules and interaction, immune disease, immune system, infectious disease: viral and it was found that DEGs was mainly concentrated in several key points (**Figure 2H**). The KEGG pathway enrichment analysis comparing the hypothyroidism group with healthy controls found that the DEGs were mainly enriched in several key pathways, including signal transduction, infectious disease: bacterial, immune disease, energy metabolism, and immune system (**Figure 2I**). We further employed KEGG enrichment scatter plots to analyze the occurrence of DEGs within specific pathways and to examine the interactions among these pathways. The analysis comparing the hyperthyroidism group with healthy controls revealed that the DEGs were mainly enriched in the following pathways: influenza A, circadian rhythm and B cell receptor signaling pathway (**Figures 2J, K**). In contrast, the comparison between the hypothyroidism group and healthy controls showed that DEGs were mainly enriched in pathways related to Th17 Cell differentiation, thermogenesis (associated with ATP production), oxidative phosphorylation, reactive oxygen species and Th1 and Th2 Cell Differentiation (**Figures 2L, M**).

### 3.3 Reactome enrichment analysis of DEGs

**Figure 3:**
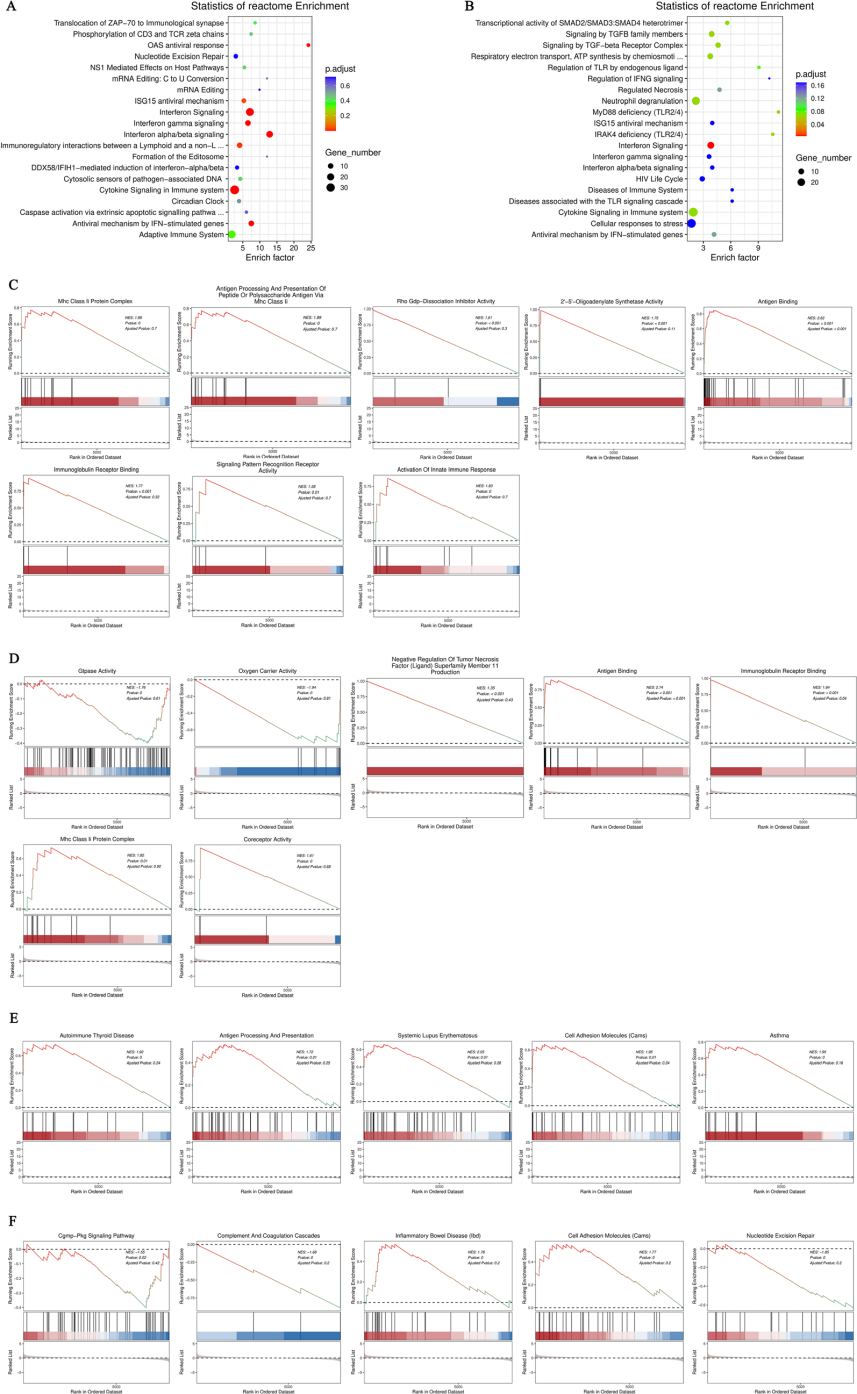
**(A)** Enrichment scatter plot of Reactome pathways for differentially expressed genes comparing the hyperthyroid group to the normal group; **(B)** Enrichment scatter plot of Reactome pathways for differentially expressed genes comparing the hypothyroid group to the normal group. In these plots, each row represents a specific Reactome pathway (Term). The x-axis indicates the enrichment factor, which is the ratio of the proportion of differentially expressed genes annotated to that Term compared to the proportion of all genes annotated to the same Term. A larger enrichment factor signifies a more significant level of enrichment of differentially expressed genes within that Term. The color of each point reflects the p.adjust value, indicating the significance of enrichment, while the size of the points corresponds to the number of differentially expressed genes annotated to that specific Term; **(C)** GO-GSEA for differentially expressed genes comparing the hyperthyroid group to the normal group; **(D)** GO-GSEA for differentially expressed genes comparing the hypothyroid group to the normal group; **(E)** KEGG-GSEA for differentially expressed genes comparing the hyperthyroid group to the normal group. **(F)** KEGG-GSEA for differentially expressed genes comparing the hypothyroid group to the normal group. The enrichment plots are divided into three sections: Upper Section - ES Line Plot: This section presents the enrichment score (ES) as a line graph, illustrating how the ES value is computed at each position along the ordered gene list during the analysis. The peak of the ES curve represents the maximum ES score, with the distance from zero indicating the significance of enrichment—the farther the peak from zero, the more significant the enrichment. Middle Section - Hit Chart (Barcode Plot): This section, commonly referred to as a hit chart or barcode plot, employs lines or “hits” to mark the positions of genes that are members of the analyzed pathways within the ranked gene list sorted by log2 fold change values (from high to low). This visual representation highlights where pathway-associated genes appear in relation to the overall ranking, providing insight into their contribution to the enrichment score. The lower section of the enrichment plot displays the distribution of rank values for all sorted genes. In this heatmap representation, the red areas correspond to genes that are highly expressed in group A (e.g., hyperthyroid), while the blue areas indicate genes that are more highly expressed in group B (e.g., hypothyroid). Each gene’s signal-to-noise ratio (Signal2noise), calculated using the previously selected ranking method (log2 fold change), is illustrated through a red/gray/blue area plot. The Enrichment Score (ES) is depicted as an accumulated value, representing the cumulative enrichment of genes from the gene set within the ranked list. The size of the ES indicates the extent of enrichment, with larger ES values reflecting a higher degree of enrichment of the gene set in the sorted list. The Normalized Enrichment Score (NES) is a value that normalizes the Enrichment Score (ES), representing the degree of enrichment of a gene set in the ranked list of genes. NES>0: Indicates that the gene set is enriched in the upper part of the ranked gene list (positive direction), suggesting that these genes tend to be upregulated under the experimental conditions being studied. NES<0: Suggests that the gene set is enriched in the lower part of the ranked gene list (negative direction), indicating that these genes are likely to be downregulated under the research conditions. NES≈0: Indicates that there is no significant enrichment of the gene set in the ranked list.

After conducting KEGG enrichment analysis on the selected DEGs, we performed a Reactome enrichment analysis to clarify cell BP, metabolic pathways, signaling pathways, and gene regulation associated with these DEGs. The outcome of the Reactome enrichment scatter plot for the hyperthyroidism group compared to the healthy controls enunciative that the DEGs were predominantly enriched in several key pathways, including cytokine signaling in immune system, OAS antiviral response, ISG15 antiviral mechanism and interferon alpha/beta signaling (**Figure 3A**). The Reactome enrichment analysis for the hypothyroidism group versus the healthy controls showed that the DEGs were primarily concentrated in the following pathways: interferon signaling, cytokine signaling in immune system, and regulation of Toll-like receptors (TLR) by endogenous ligand (**Figure 3B**).

### 3.4 Gene database annotation analysis

Gene Set Enrichment Analysis (GSEA) is a powerful method for identifying gene sets associated with specific biological processes, pathways, or phenotypes. To further investigate the significant impacts of biological functions on gene expression revealed in the GO analysis, we conducted GO-GSEA, allowing us to uncover changes and regulatory mechanisms in cellular functions. In the comparison between the hyperthyroidism group and the healthy controls, the following biological processes were positively correlated with differential gene expression: antigen binding, immunoglobulin receptor binding, activation of innate immune responses, MHC class II protein complexes, antigen binding, antigen presentation, 2’-5’ oligoadenylate synthetase activity, Rho GDP-dissociation inhibitor activity and MHC class II antigen processing (**Figure 3C**).

In the GO-GSEA analysis comparing the hypothyroidism group with the healthy controls, the results indicated that antigen binding, co-receptor activity, immunoglobulin receptor binding were negatively correlated with differential gene expression. Conversely, GTPase activity, oxygen carrier activity, MHC class II protein complexes, and TNFSF 11 were positively correlated with differential gene expression (**Figure 3D**). The KEGG-GSEA analysis revealed that antigen processing and presentation, autoimmune thyroid disease, cell adhesion molecules, systemic lupus erythematosus were significantly affected in the hyperthyroidism group (**Figure 3E**). In contrast, complement and coagulation cascades, cell adhesion molecules (CAMs), inflammatory bowel disease (IBD), nucleotide excision repair and cGMP-PKG signaling pathway, were significantly impacted in the hypothyroidism group (**Figure 3F**).

### 3.5 Gene CIBERSORT and ssGSEA analysis

**Figure 4:**
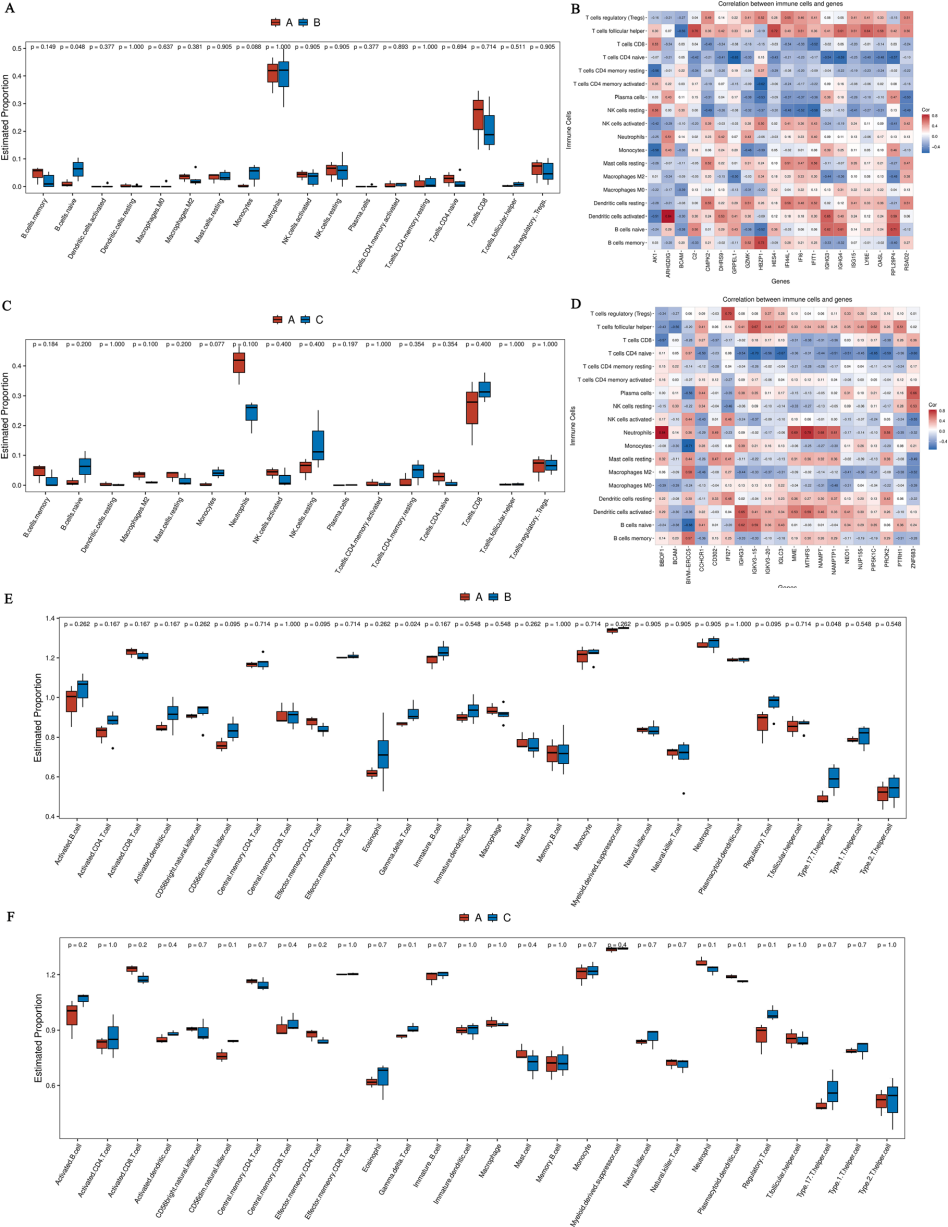
**(A)** Boxplot comparing the immune cell annotations between the hyperthyroid group and the normal group. The x-axis represents different types of immune cells, while the y-axis indicates the proportion of each immune cell type; **(B)** Correlation analysis between core differentially expressed genes and the proportions of immune cells in the hyperthyroid group compared to the normal group; **(C)** Boxplot comparing the immune cell annotations between the hypothyroid group and the normal group. Similar to Panel A, the x-axis indicates various immune cell types, and the y-axis shows their respective proportions; **(D)** Correlation analysis between core differentially expressed genes and the proportions of immune cells in the hypothyroid group compared to the normal group. The x-axis represents the differential genes (the top 20 differential genes with the minimum q-value and the largest absolute log2 fold change), while the y-axis denotes cell types, with different colors indicating the magnitude of correlation; **(E)** The boxplot of ssGSEA annotations for genes comparing the hyperthyroid group to the normal group; **(F)** The boxplot of ssGSEA annotations for genes comparing the hypothyroid group to the normal group. The x-axis refers to cell types, and the y-axis indicates the relative abundance of different cell types.

We utilized the CIBERSORT computational biology tool to estimate the relative proportions of diverse immune cell types. The analysis inferred the presence and relative plenty of varieties of immune cells from changes in differential genes, as well as the relationship between quick differential genes and the proportions of immune cells. Comparing the hyperthyroidism group with the healthy controls revealed significant differences in the following immune cell types: naive B cells, monocytes, naive CD4^+^T cells, and Tregs (**Figures 4A, B**). The comparison between the hypothyroidism group and the healthy controls showed valid differences in the following immune cell types: naive CD4^+^T cells, naive B cells, CD8^+^T cells and memory B cells (**Figures 4C, D**).

Using ssGSEA to break down gene expression data from individual samples, we evaluated the activity levels of specific gene sets within each sample. In the comparison between the hyperthyroidism group and the healthy controls, the following immune cell types exhibited significantly increased activity: immature B cells, activated CD4^+^T cells, activated CD8^+^T cells, CD56dim natural killer cells, gamma delta T cells, effector memory CD4^+^T cells and Tregs (**Figure 4E**). In contrast, the comparison between the hypothyroid patients and the healthy person revealed significantly elevated activity levels in CD8^+^T cells, CD56dim natural killer cells, effector memory CD4^+^T cells, gamma delta T cells and central memory CD8^+^ T cells (**Figure 4F**).

### 3.6 Differential protein screening

**Figure 5:**
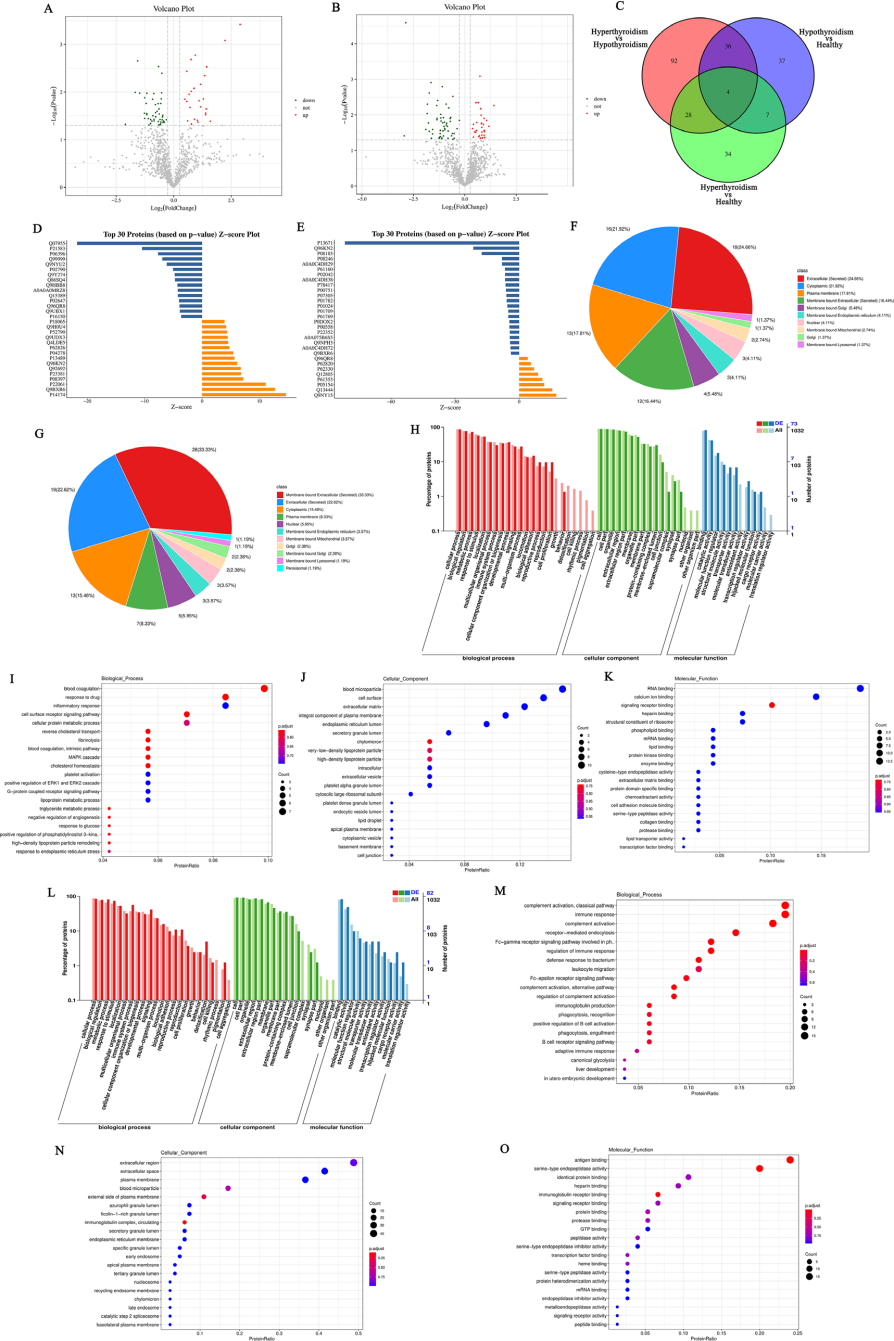
**(A)** Volcano plot of differentially expressed proteins between the hyperthyroid group and the normal group; **(B)** Volcano plot of differentially expressed proteins between the hypothyroid group and the normal group. Each point in the figure represents a protein, with the x-axis indicating the logarithmic value of the fold change in expression levels between the two samples, and the y-axis representing the negative logarithmic value of the p-value. A larger absolute value on the x-axis indicates a greater fold change in expression levels between the two samples, while a higher value on the y-axis signifies more significant differential expression, thereby enhancing the reliability of the identified differentially expressed proteins. Green points in the plot represent downregulated differentially expressed proteins, red points indicate upregulated differentially expressed proteins, and black points denote non-differentially expressed proteins; **(C)** Venn diagram of differentially expressed proteins: The shapes of different colors represent various comparison groups, and the numbers in the overlapping areas indicate the number of shared differentially expressed proteins between two comparison groups. The numbers in the overlapping sections among multiple colored shapes represent the count of differentially expressed proteins common to those multiple comparison groups; **(D)** Histogram of Z-scores for differentially expressed proteins between the hyperthyroid group and the normal group; **(E)** Histogram of Z-scores for differentially expressed proteins between the hypothyroid group and the normal group. The x-axis represents the Z-score values, while the y-axis indicates the differentially expressed proteins. The Z-score is calculated using the mean and standard deviation of the experimental group compared to the control group; a value further to the right indicates a higher relative abundance of the protein in the experimental group; **(F)** Subcellular localization of differentially expressed proteins comparing the hyperthyroid group with the normal group; **(G)** Subcellular localization of differentially expressed proteins comparing the hypothyroid group with the normal group; **(H)** Statistical classification chart of GO annotations for differentially expressed proteins between the hyperthyroid group and the normal group. The x-axis represents the GO categories, while the left y-axis indicates the percentage of proteins, and the right y-axis shows the absolute number of proteins; **(I)** Scatter plot of GO annotations for BP comparing hyperthyroid vs. normal differentially expressed proteins; **(J)** Scatter plot of GO annotations for CC comparing hyperthyroid vs. normal differentially expressed proteins; **(K)** Scatter plot of GO annotations for MF comparing hyperthyroid vs. normal differentially expressed proteins; **(L)** Statistical classification chart of GO annotations for differentially expressed proteins between the hypothyroid group and the normal group. The x-axis represents the GO categories, while the left y-axis indicates the percentage of proteins, and the right y-axis shows the absolute number of proteins; **(M)** Scatter plot of GO annotations for BP comparing hypothyroid vs. normal differentially expressed proteins; **(N)** Scatter plot of GO annotations for CC comparing hypothyroid vs. normal differentially expressed proteins; **(O)** Scatter plot of GO annotations for MF comparing hypothyroid vs. normal differentially expressed proteins.

Considering the time and spatial specificity of protein expression, we conducted differential protein analysis on serum samples from patients in the hyperthyroid group, hypothyroid group, and normal group. The results showed that there was a total of 73 differentially expressed proteins (DEPs) among the hyperthyroid group and the normal group, with 26 proteins upregulated and 47 proteins downregulated (**Figure 5A**). In the hypothyroid group and the normal group, the sum of all 84 DGPs were confirmed, various 30 proteins that were up-regulation and 54 proteins that were downregulated (**Figure 5B**). Furthermore, we created Venn diagrams to illustrate multitude unique distinguishingly expressed proteins in several group as well as the shared differentially expressed proteins across groups (**Figure 5C**).

### 3.7 Differential protein Z-score analysis and subcellular localization

We utilized the Z-score (standard score) to measure the relative abundance of proteins in the hyperthyroidism group contrapositive to the healthy people. **Figure 5D** presents the Z-score values of the top 30 DEPs, sorted by p-value. Additionally, **Figure 5E** illustrates the Z-score values for DEPs in the hypothyroidism group relative to the healthy controls.

In order to understand the various cellular functions of proteins, it is first necessary to clarify their subcellular positioning, as proteins must be transported to the correct positioning within the cell to participate in biological activities. Therefore, we analyzed the appeals screen for subcellular positioning of proteins. In the comparison among the hyperthyroidism group and the healthy controls, the results of the subcellular positioning analysis indicated that 24.66% of the DEPs were classified as extracellular (secreted), 21.92% as cytoplasmic, 17.81% as plasma membrane, and 16.44% as membrane-bound extracellular (secreted) (**Figure 5F**). For the hypothyroidism group compared to the healthy controls, the subcellular positioning analysis revealed that 33.33% of the DGPs were membrane-bound extracellular (secreted), 22.62% were extracellular (secreted), 15.48% were cytoplasmic, and 8.33% were plasma membrane (**Figure 5G**).

### 3.8 Protein functional annotation results

After determining the subcellular positioning of the proteins, we performed them with GO enrichment analysis to examine the distribution of these proteins and clarify how the variations observed in our experimental samples are reflected in their functional roles. In the comparison between the hyperthyroidism group and the healthy controls, DEPs functions were evident across several BP, including: immune system process, cell surface receptor signaling pathway, metabolic process, biological adhesion and cell proliferation. The analysis also revealed significant MF associated with these proteins, such as: signaling receptor binding, molecular function regulator, transporter activity, molecular transducer activity, transcription regulator activity and transporter activity. Additionally, regarding CC, the enriched categories included: synapse part, cell junction, supramolecular complex, synapse part, organelle part and membrane-enclosed lumen (**Figures 5H–K**). The DEPs functions between the hypothyroidism group and the healthy controls were reflected in several BP, including immune response, receptor−mediated endocytosis, complement activation, classical pathway and B cell receptor signaling pathway. Notably, the following MF were significantly enriched: molecular function regulator, antigen binding, immunoglobulin receptor binding, catalytic activity, antioxidant activity and structural molecule activity. Additionally, in CC that exhibited differences included immunoglobulin complex, circulating, membrane part, protein-containing complex and cell junction (**Figures 5L–O**).

**Figure 6:**
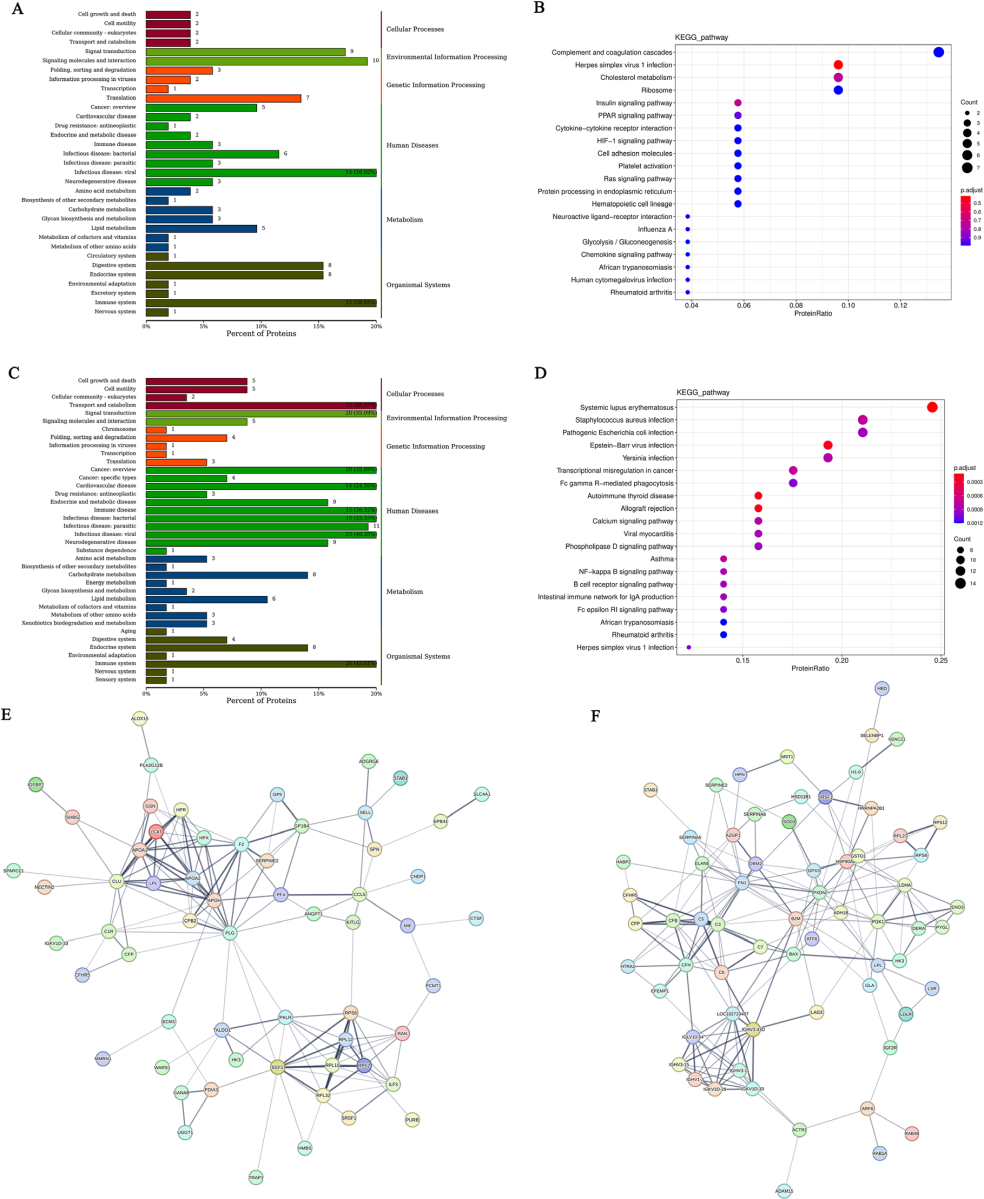
**(A)** KEGG classification chart of differentially expressed proteins between the hyperthyroid group and the normal group; **(C)** KEGG classification chart of differentially expressed proteins between the hypothyroid group and the normal group. The y-axis represents the names of KEGG secondary metabolic pathways, while the x-axis denotes the number of proteins annotated to each pathway and the proportion of these proteins relative to the total number annotated. **(B)** Scatter plot of KEGG pathway enrichment for differentially expressed proteins comparing the hyperthyroid group with the normal group; **(D)** Scatter plot of KEGG pathway enrichment for differentially expressed proteins comparing the hypothyroid group with the normal group. In the figure, each row represents a KEGG pathway. The x-axis indicates the enrichment factor, which is the ratio of the proportion of differentially expressed proteins annotated to that pathway to the proportion of all proteins annotated to that pathway. A larger enrichment factor indicates a more significant level of enrichment of differentially expressed proteins in that pathway. The color of the points represents the q-value, while the size of the points reflects the number of differentially expressed proteins annotated to that particular pathway; **(E)** Interaction network diagram of differentially expressed proteins between the hyperthyroid group and the normal group; **(F)** Interaction network diagram of differentially expressed proteins between the hypothyroid group and the normal group. Each node represents a protein, and the thickness of the lines indicates the confidence level of the associations, with thicker lines representing higher confidence in the interactions.

The KEGG annotation of DEPs revealed significant impacts on various signaling pathways when comparing the hyperthyroidism group to the healthy controls. Notable pathways include herpes simplex virus 1 infection, insulin signaling pathway, complement and coagulation cascades and cytokine-cytokine receptor interaction (**Figures 6A, B**). In contrast, the comparison between the hypothyroidism group and the normal healthy controls highlighted significant effects on NF-kappaβ signaling pathway, disease-related signaling pathways, including systemic lupus erythematosus, autoimmune thyroid disease and B cell receptor signaling pathway (**Figures 6C, D**).

To elucidate how proteins interact with one another and their role in various BP such as signal transduction, energy and material metabolism, cell cycle control and gene expression regulation. we employed the STRING dbase along with the StringDB protein reciprocity database to conduct a decomposition of protein interactions specific to the relevant species. The results of the differential protein interaction network for the hyperthyroidism group compared to the healthy controls are displayed in **Figure 6E**, while the differential protein interaction network for the hypothyroidism group versus the healthy controls is shown in **Figure 6F**.

### 3.9 Conjoint analysis of transcriptomics and proteomics

**Figure 7:**
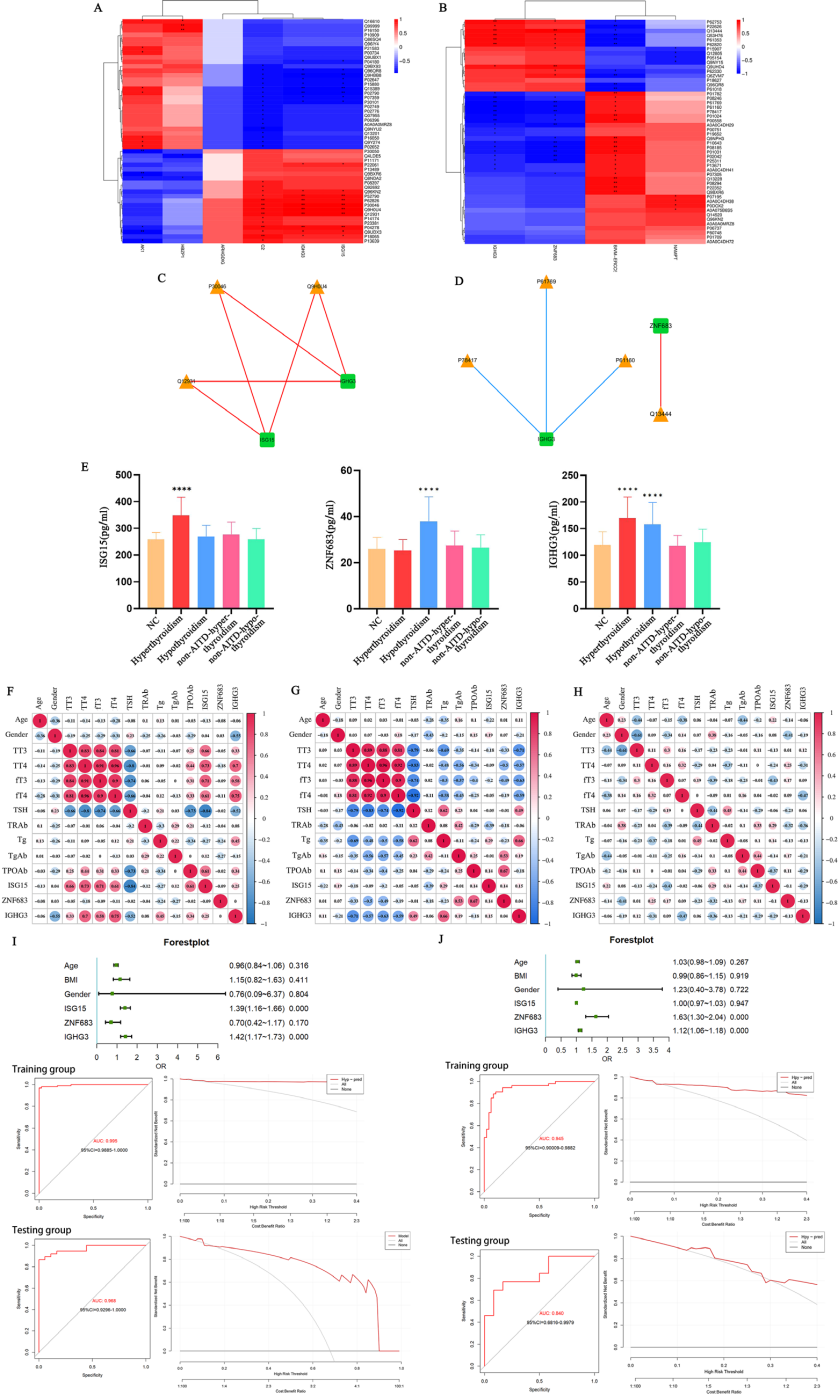
**(A)** Spearman correlation heatmap of differentially expressed genes and proteins between the hyperthyroid group and the normal group; **(B)** Spearman correlation heatmap of differentially expressed genes and proteins between the hypothyroid group and the normal group. In the heatmap, the columns represent differentially expressed genes, and the rows represent differentially expressed proteins. The strength of the correlation is indicated by varying colors. *p<0.05, **p<0.01; **(C)** Network regulatory diagram comparing the hyperthyroid group with the normal group; **(D)** Network regulatory diagram comparing the hypothyroid group with the normal group. In the diagrams, rectangles represent differentially expressed genes, and triangles represent differentially expressed proteins. Blue lines indicate negative correlations, while red lines indicate positive correlations; **(E)** Levels of ISG15, ZNF683, and IGHG3 in the serum of hyperthyroid patients, hypothyroid patients, and healthy individuals. ****P<0.001; **(F)** Correlation of IGHG3, ISG15, and ZNF683 with clinical examination data in hyperthyroid patients; **(G)** Correlation of IGHG3, ISG15, and ZNF683 with clinical examination data in hypothyroid patients; **(H)** Correlation of IGHG3, ISG15, and ZNF683 with clinical examination data in healthy individuals; **(I)** Clinical prediction models for patients with hyperthyroidism and ROC curves (left) and DCA (right) for training and test groups; **(J)** Clinical prediction model for patients with hypothyroid and ROC curves (left) and DCA (right) for training and test groups. DCA Baseline: Represents the decision-making performance without using a predictive model. If the decision curve of the predictive model lies above the baseline, it indicates that the model makes a positive contribution to the decision, providing better decision outcomes. Decision Curve of the Predictive Model: By examining the decision curve of the predictive model, one can observe how the model’s performance changes under different decision thresholds. The closer the decision curve is to the baseline, the poorer the predictive model’s effect on decision-making; conversely, the further the decision curve is from the baseline, the better the model’s contribution to decision-making.

In this research, we implemented a conjoint analysis of transcriptomics and proteomics. We selected the top 50 DEPs with the lowest FDR from the transcriptomic analysis and the top 50 proteins with the lowest significance *P*-values from the proteomic analysis for correlation analysis. Using the selected Top 50 DEGs and Top 50 DEPs, we calculated the Spearman correlation between the two datasets (**Figures 7A, B**). Furthermore, we filtered for results with an absolute value>0.9 and *P*<0.001, which were used to construct a regulatory network diagram. The correlation results obtained were visualized using Cytoscape software, resulting in a regulatory network diagram that allowed us to identify the most critical differential genes (**Figures 7C, D**).

### 3.10 ELISA analysis of serum results in clinical subjects

Using the ELISA method, we assessed the expression levels of IGHG3, ISG15, and ZNF683 in the serum of all participants. The consequences indicated that the serum content of IGHG3 were meaningfully higher in both AITD hyperthyroidism and hypothyroidism group patients contrapositive to the healthy controls and non-AITD patients. Furthermore, compared to healthy individuals or patients with non-AITD conditions, only the hyperthyroid group of patients with AITD exhibited elevated serum levels of ISG15. Additionally, only the hypothyroid group of patients with AITD showed increased serum levels of ZNF683 (*P*<0.05), (**Figure 7E**).

The expression levels of IGHG3, ISG15, and ZNF683 were analyzed in relation to clinical examination data from patients with hyperthyroidism, hypothyroidism, and healthy individuals. The results indicated that IGHG3 expression was positively correlated with the levels of TT3, TT4, fT3, fT4, and Tg in patients with AITD. Additionally, ISG15 expression showed a positive correlation with TT3, TT4, fT3, and fT4 levels in AITD hyperthyroid patients, while exhibiting a negative correlation with TSH levels. Conversely, ZNF683 expression was negatively correlated with the levels of TT3, TT4, fT3, and fT4 in AITD hypothyroid patients, and positively correlated with TgAb and TPOAb expression levels (**Figures 7F–H**).

### 3.11 Construction of predictive models for hyperthyroidism and hypothyroidism

Using clinical baseline data collected from participants and ELISA results for IGHG3, ISG15, and ZNF683, we randomly divided the samples into training and testing groups to construct prediction models for patients with AITD hyperthyroidism and hypothyroidism. Forest plots were created, ROC curves and decision curve analysis (DCA) were hatched to evaluate the performance and decision effect of these models. The results showed that the prediction model of AITD hyperthyroidism patients had good performance and decision-making effect (**Figure 7I**). The predictive model for AITD hypothyroidism patients had good performance and decision-making effect (**Figure 7J**). These results indicate that both predictive models have significant utility in predicting hyperthyroidism or hypothyroidism in patients with AITD.

## 4 Discussion

AITD represent a category of organ-specific autoimmune disorders, characterized by thyroid dysfunction primarily resulting from the outcome of thyroid-specific antibodies by the immune system. This dysregulation leads to either stimulation or destruction of the thyroid gland, as well as an imbalance in self-tolerance to autoantibodies. The most prevalent forms of AITD involve Graves’ disease (GD) and Hashimoto’s thyroiditis (HT), with clinical manifestations corresponding to hyperthyroidism and hypothyroidism, respectively (9). GD is recognized as the primary career of hyperthyroidism, with its pathogenesis predominantly involving the activation of immunoglobulin G subclass antibodies against the thyroid-stimulating hormone receptor (TRAb) (10). This condition is often linked with T cell disfunction, disruption of immune tolerance, aberrant immune regulation, and increased B cell reactivity, ultimately resulting in damage to the thyroid and associated tissues (11). A hallmark for the diagnosis of GD is the presence of elevated TRAb levels in serum, which is observed in approximately 97-98% of cases. HT is commonly recognized as the major career of hypothyroidism (12, 13). As major prevalent autoimmune shambles, HT is described by the production of thyroid-specific autoantibodies and the infiltration of T cells and B cells in inflammatory processes. Both cellular immunity and humoral immunity act pivotal characters its pathogenic mechanism (4). Presently, the diagnosis of HT primarily relies on clinical features, positive serum levels of TPOAb and TgAb, along with cytological examination revealing lymphocytic infiltration. In clinical practice, patients with AITDs often present with subtle or non-specific symptoms in the early stages of the disease, making early detection challenging. Furthermore, these patients frequently experience transitions between hyperthyroidism and hypothyroidism, yet there is currently no reliable method to predict or assess these two disease states. Previous studies have suggested using the ratio of TT3/TT4 or fT3/fT4 to differentiate between GD and HT; however, this approach has demonstrated insufficient specificity (1). The precise etiology of AITDs has yet to be fully elucidated; however, their onset and progression are believed to be associated with the interplay of genetic factors, environmental influences, and epigenetic modifications. The molecular mechanisms underlying immune dysfunction that led to the breaking of thyroid tissue remain mostly unclear (14). Therefore, a comprehensive understanding the mechanism of AITD development is very important for clear and early diagnosis and clinical individualized treatment. This study employs multi-omics techniques, including gene transcriptomics and proteomics, to shift from superficial routine indicators to in-depth investigations at the gene and protein levels. The aim is to elucidate the emergence and advancement of AITD and to identify reliable marker for the early diagnosis and differential diagnosis of clinical patients. It is anticipated that this research will afford an element for the diagnosis and individualized therapy of AITD patients.

Graves’ disease is triggered by the decrease of immune tolerance to the thyroid-stimulating hormone receptor (TSHR), with its specificity and core mechanism centered on the activation of immunoglobulin G subclass antibodies against TSHR (TRAb). TRAb bind to the leucine-rich repeat region situated in the extracellular domain of TSHR on the surface of thyroid cells (15). The immune response induced by the specific binding of antigen receptors to autoantibodies is fundamental to the pathogenesis of this disease. Hence, we utilized blood samples from patients with primary hyperthyroidism in clinical settings to conduct gene transcriptomic analysis for differential gene functional annotation and enrichment analysis. The consequences indicated that DEGs were primarily enriched in BP related to immune response and immune response-activation signaling pathways. Proteomic analysis showed that DEPs were mainly enriched in BP such as cell surface receptor signaling pathways, the immune system, and cell proliferation. In terms of molecular functions, these proteins showed enrichment in antigen binding, immunoglobulin binding, IgG receptor activity, and signaling receptor binding. The cellular component analysis indicated a primary enrichment in locations such as the cell surface, membrane side, and extracellular membrane. Additionally, proteomic analysis identified subcellular localization of DEPs predominantly in the extracellular (secreted) compartment (24.66%), cytoplasm (21.92%), and membrane-associated extracellular (secreted) regions (16.44%). Trough KEGG-GSEA enrichment analysis, we found that differentially expressed genes were significantly associated with autoimmune thyroid diseases, Systemic lupus erythematosus (SLE), antigen processing and presentation rheumatoid arthritis (RA). This suggests that these differential genes have an important effect on the pathogenesis of autoimmune diseases. Furthermore, studies have reported an association between systemic autoimmune diseases (such as Sjogren’s syndrome, RA, SLE) and AITD (12), indicating a notable effect of these DEGs in the occurrence and progression of AITDs.

AITD is characterized as an organ-specific autoimmune disease arbitrated by both B cells and T cells. Our KEGG annotation analysis revealed an enrichment in the B cell receptor signaling pathway, highlighting the importance of this pathway in the underlying mechanisms of AITDs. According to the expression of DEGs, we inferred the presence and relative enrichment of various immune cell types. Our study results indicated significant differences in naive B cells, monocytes, follicular helper T cells, Tregs and naive CD4^+^T cells. Furthermore, research has shown that patients with GD exhibit an abnormal enhancement in CD4^+^T cells and CD8^+^T cells (16, 17), underscoring the central role of B cells and T cells in the pathogenic mechanism of AITD. Subsequently, we conducted a joint analysis of transcriptomics and proteomics to examine the relationship among differential genes and proteins, as well as to analyze the common pathways and regulatory networks involving these differential genes and proteins. In the regulatory network diagram we obtained, we identified that the major differential genes were ISG15 and IGHG3. These findings suggest potential key players in the immune response related to AITD and may provide insights for further investigation into therapeutic targets and biomarkers for this condition.

ISG15 is a member of the interferon-stimulated genes (ISGs) family and the first ubiquitin-like protein to be identified. Its functions involve viral replication, cell proliferation, cell cycle regulation, DNA damage repair, protein translation, immune regulation and many other directions (18). As most important robustly and rapidly persuaded type I ISGs, ISG15 exerts its effects through covalent binding to target proteins, leading to ISGylation, which directly inhibits protein replication and modulates host immune responses. The ISG15 released by immune cells function in immune regulation (19); it can function as a warning protein that activates CD8^+^T cells and triggers NK cell excretion of IFN-γ in conjunction with IL-12 via the LFA-1 receptor (20). ISG15 is persuaded by type I interferons, and research have given that ISG15 is extremely expressed in vitiligo. In the existence of IL-15, ISG15 significantly induces the excretion of IFN-γ, contributing to the pathogenesis and progression of vitiligo, ultimately leading to melanocyte damage (21). Raised criterion of ISG15 has been observed in the saliva and serum of sufferers with major Sjogren’s syndrome, and ISG15 expression is also relatively high in SLE sufferers, correlating with disease progression prior to treatment. Research indicates that ISG15 serves as a common central gene in both diseases (22). Moreover, studies have found that patients with high ISG expression are more applicable to present rash-related symptoms contrapositive to those with low expression levels (23). Among SLE sufferers who are active for antinuclear antibodies (ANA), anti-chromatin, anti-Smith, or anti-C1q antibodies, overexpression of ISG15 is more prevalent, and the level of ISG15 expression positively correlates with ANA and anti-dsDNA titers (24). In this research, we employed a combined decomposition of gene transcriptomics and proteomics to recognize ISG15 as a differential gene unique to patients with primary hyperthyroidism compared to healthy individuals. We observed an enhancement expression of ISG15 in the serum of hyperthyroidism patients, indicating a significant correlation between ISG15 and GD. Elisa assays showed that the serum levels of ISG15 were expressively upper in the hyperthyroid group contrapositive to both the hypothyroid group and the normal control group, with no valid difference scanning among the hypothyroid and normal groups. Subsequently, we regulated a correlation analysis among representative of ISG15 and clinical blood parameters as well as fundamental characteristics in sicks with hyperthyroidism. Our findings demonstrated that ISG15 expression was specifically correlated with the quantity of TT3, TT4, fT3, and fT4, while layout a passive correlation with TSH levels. These results suggest that elevated ISG15 expression a lot of invites as a pointer of hyperthyroid status in patients. There are still delays in detection and treatment of changes in patients with hyperthyroidism or their potential progression to hypothyroidism due to changes in TRAb activity that are not detected in a timely manner. Our research highlights the specific high expression of ISG15 in hyperthyroidism patients and its correlation with thyroid function, suggesting that ISG15 could serve as a diagnostic biomarker for hyperthyroidism. This discovery provides an element for understanding the pathogenesis of AITD in hyperthyroidism patients and for facilitating individualized diagnosis and treatment approaches. In our analysis of gene transcriptomics comparing patients clinically diagnosed with primary hypothyroidism to healthy individuals, we conducted functional annotation and enrichment analysis of the DEGs. The results indicated that these genes were primarily enriched in pathways participant to immune response regulation, autophagy, immunoglobulin complexes, particularly IgG immunoglobulin complexes and B-cell receptor signaling. The KEGG analysis showed valid enrichment in processes like Th1 and Th2 cell differentiation, thermogenesis, oxidative phosphorylation, reactive oxygen species (ROS) production, Th1 and Th2 cell differentiation. Previous studies have suggested that Th17 cell infiltration in the thyroid can significantly increase serum IL-17 levels in HT patients. This imbalance in the differentiation of peripheral blood mononuclear cells towards Th1 and Th17 subtypes could be linked with the pathogenic mechanism of HT (25, 26). Reactive oxygen species are elementary to the criterion revolution of thyroid follicular cells. Research conducted in mouse and human models has shown that ROS can lead to apoptosis of thyroid cells, linking them to the pathogenesis of HT (27). In our study of HT patients utilizing gene transcriptomics, we also validated the presence and relative abundance of different immune cell types, identifying significantly active populations such as activated CD8^+^ T cells, CD56dim natural killer cells, central memory CD8^+^ T cells, effector memory CD4^+^ T cells, and gamma delta T cells. Notably, it has been observed that there is an abnormal increase in CD4^+^ T cells, CD8^+^ T cells, and macro TPOAb ages within HT (16, 17). These discoveries highlight the sophisticated interaction effect among immune cell dynamics and thyroid function in the context of primary hypothyroidism, suggesting potential therapeutic targets for managing autoimmune thyroid conditions. Therefore, the cellular immune processes mediated by CD4^+^ T and CD8^+^ T lymphocyte subpopulations have an important role in the pathogenesis of HT. Proteomic analysis revealed that the functional annotation of DEPs was primarily enriched in pathways related to the NF-κβ signaling pathway, systemic lupus erythematosus, autoimmune thyroid diseases and the B cell receptor signaling pathway. Through a combined analysis of gene transcriptomics and proteomics between the hypothyroidism group and healthy controls, we identified key differential genes ZNF683 and IGHG3 in the resulting network regulatory diagram.

ZNF683 is a transcription factor that regulates gene expression. Its homologous gene, Blimp-1, shares a similar gene structure, both containing zinc finger domains (28). Consequently, ZNF683 is referred to as HOBIT, representing the homolog of BLIMP-1 in T cells. ZNF683 is a key regulatory factor in the early variation of human NK cells. It is also both inevitable and competent for the outcome of IFN-γ by CD8^+^T cells, promoting the variation of lymphocytes into long-lived effectors in non-lymphoid organs and other non-barrier tissues, thereby supposing instant immune safeguard in opposition to reinfection. ZNF683 enhances the proliferative capacity and IFN-γ secretion of CD8^+^T cells. When ZNF683 is knocked out in human peripheral blood mononuclear cells, the diffusion of CD8^+^T cells is modest, and the outcome of IFN-γ is reduced (29). Research have given that high expression of ZNF683 facilitate the proliferation and excretion of IFN-γ by CD8^+^T cells following infection. IFN-γ plays an important role in managing viral replication and microbial invasion, which may lead to sustained inflammatory responses and disease progression. These consequences suggest that ZNF683 could serve as a biomarker for CD8^+^T cell action and may be linked with disease development (30). Furthermore, it has been reported that TBX21 is one of the upstream genes of ZNF683. Due to its expression of IFN-γ, TBX21 significantly impacts other immune cells, including Th1 cells (31). ZNF683 is a crucial gene that controls the variation and activation of T cells, serving as a key regulatory factor in controlling the status and activation of human T cells. ZNF683 can target various genes and pathways associated with T cell induction, effector functions, activation, and cytotoxicity. Research has demonstrated that ZNF683^+^CD8^+^T cell totality is important for anti-tumor immunity, with high levels of this ZNF683 gene marker detected in peripheral blood samples from patients with renal cell carcinoma, melanoma, and lung cancer (32). In our research, we found elevated quantity of ZNF683 expression in the serum of hypothyroid patients. Correlation analyses between the expression of ZNF683 and clinical blood test indicators as well as basic clinical characteristics in patients with hyperthyroidism revealed that ZNF683 expression negatively correlated with the levels of TT3, TT4, fT3 and fT4, while positively correlating with the levels of TgAb and TPOAb. This suggests that high expression of ZNF683 may indicate that patients are in a state of hypothyroidism, highlighting ZNF683 as an important risk factor for the progress of this condition. Moreover, it is hypothesized that ZNF683 may make contribution to the beginning of hypothyroidism through the modulation of immune responses mediated by CD8^+^ T cells. These findings afford an element for further research into the organs underlying the progress of hypothyroidism and suggest that ZNF683 could serve as one of the diagnostic biomarkers for this condition. This information may offer new insights for personalized treatment strategies in patients with AITD and hypothyroidism.

Human antibodies, or immunoglobulins, were initially defined as γ-globulins. IgG is classified into four subgenera: IgG1, IgG2, IgG3, and IgG4. IGHG3 represents the permanent area of the immunoglobulin heavy chain. It is a glycoprotein produced by B lymphocytes that can exist in either a membrane-bound or secreted form. This subclass is characterized by a slender hinge region, which provides biggish flexibility and features attached glycosylation sites, allowing the antibody effector capabilities to be adjusted by the extent of the IgG3 hinge region (33). Research conducted by Chu et al. on hinge variants in the IGHG3 isotype backbone revealed that a reduction in hinge length resulted in decreased phagocytic activity of IgG3 (34). As an effective immunoglobulin, IGHG3 can activate pro-inflammatory signaling through the Fc portion of IgG particles on immune cells, leading to complement activation, antibody-mediated phagocytosis, antibody- servient cellular cytotoxicity, and the production of IFN (35). Furthermore, studies indicate that by analyzing changes in the protein composition of saliva from patients with SLE, potential salivary biomarkers for SLE can be identified, revealing elevated levels of several peptides, including the constant region of IGHG3 in the slabber of SLE patients (36). Notably, enhanced levels of IGHG3 have also been observed in the saliva, serum, and urine of SLE patients, and the measurement of IGHG3 levels in urine may aid in differentiating active nephritis (33). Through our research, we found that the differentially expressed genes in both hyperthyroid and hypothyroid patients included IGHG3. Additionally, ELISA assays revealed that IGHG3 is extremely expressed in the serum of patients with hyperthyroidism and hypothyroidism. Correlation analyses indicated a strong association between IGHG3 levels and the expression levels of TT3, TT4, fT3, fT4, and Tg in these patients, suggesting that elevated IGHG3 expression is an important marker for the occurrence AITD. This finding highlights IGHG3 as a significant biomarker for the early diagnosis and assessment of disease status in AITD patients, providing new targets for subsequent research and clinical management of AITD.

## 5 Conclusions

In this study, we conducted integrated analysis of gene transcriptomics and proteomics in the hyperthyroidism group, hypothyroidism group, and healthy control group. We identified key differentially expressed genes ISG15, ZNF683, and IGHG3, and validated the specific expression of these three indicators in serum with their correlation to thyroid function. Our study indicates that ISG15 can serve as a diagnostic biomarker for hyperthyroidism, ZNF683 can be considered as one of the diagnostic biomarkers for hypothyroidism, and elevated expression of IGHG3 suggests abnormal thyroid function in patients. This finding provides new insights into the pathogenesis of autoimmune thyroid diseases and promotes personalized diagnostic and therapeutic approaches.

## Data Availability

The data used to support the results of this study were uploaded to the NCBI database with code PRJNA1198793. The clinical data supporting the results of this study are available from the corresponding author upon request.
